# Does HIV-Related Stigma Depress Social Well-Being of Youths Affected by Parental HIV/AIDS?

**DOI:** 10.3389/fpsyt.2022.898543

**Published:** 2022-06-23

**Authors:** Yafei Zhang, Jiaojiao Wan, Lili Ji, Gaigai Liu, Yixin Shi, Junfeng Zhao, Xiaoming Li

**Affiliations:** ^1^School of Psychology, Institute of Behavior and Psychology, Henan University, Kaifeng, China; ^2^Department of Health Promotion, Education, and Behavior, University of South Carolina, Columbia, SC, United States

**Keywords:** perceived social support, social trust, social well-being, perceived stigma, enacted stigma, youths affected by parental HIV/AIDS, moderated mediation effect

## Abstract

Parental illness or death due to HIV/AIDS has long-term impacts on children’s social well-being, potentially challenging the children’s basic developmental needs and future. Based on the theoretical model of social well-being, the present study tested a moderated mediation model that HIV-related stigma moderated the mediating role of social trust on the relationship between perceived social support (PSS) and social well-being. A sample of 297 youths aged 20–30 years affected by parental HIV/AIDS (57.2% male), including 129 (43.40%) AIDS orphans and 168 vulnerable youths (56.60%) completed questionnaires of perceived social support, social well-being, social trust, and HIV-related stigma. IBM SPSS 25.0 was used to conduct descriptive statistics and multiple regressions. Results showed that the mean score of PSS was 61.34 (SD = 13.99), social well-being was 57.33 (SD = 10.15), social trust was 56.21 (SD = 11.55), perceived stigma was 64.44 (SD = 16.72), and enacted stigma was 21.91 (SD = 9.73) among youths affected by parental HIV/AIDS and the PSS could predict increasing social well-being via increasing social trust. Moreover, the positive influence of PSS on social trust was moderated by the enacted stigma (*p* = 0.03), in which the positive influence was stronger among youths affected by parental HIV/AIDS who perceived or experienced low enacted stigma than those who perceived or experienced high enacted stigma. The positive impact of social trust on social well-being was moderated by perceived stigma (*p* = 0.04), in which the positive impact was more significant among youths affected by parental HIV/AIDS who perceived or experienced high perceived stigma than those who perceived or experienced low perceived stigma. These findings explained how and when the PSS affected social well-being and contributed toward an understanding of the experiences and perceptions of HIV-related stigma among youths affected by parental HIV/AIDS. This understanding may inform future research and policies toward improving the social well-being of youths affected by parental HIV/AIDS. The study also highlighted the importance of strengthening interventions on social relations and reducing HIV-related stigma for them.

## Introduction

HIV/AIDS has been identified as a serious global public health concern. In 2020, around 37.7 million people were living with HIV/AIDS globally, of which China had about 1.053 million people, and 351,000 cumulative reported deaths (1, 2). Despite China has carried out a series of interventions (2), the HIV/AIDS epidemic has had a detrimental effect on people living with HIV/AIDS and their family members, including children and youths. Youths (aged 20–30) affected by parental HIV/AIDS, including youths who lost one or both of their parents to HIV/AIDS (“AIDS orphans”) and youths who are living with HIV-infected parents (“Vulnerable children”) (3, 4). Previous research has focused on the effects of risk factors on the negative psychosocial adjustment (depressive symptoms, problem behaviors, etc.) of children affected by parental AIDS, including loneliness (5), childhood adversity (6), peer victimization (7), HIV-related stigma (8, 9) and their interactive effects (10), etc. Therefore, based on the Chinese government’s policy of caring for vulnerable groups, as well as the support and intervention of previous researchers and social institutions (11), the existing social support and the positive psychology context, how to promote their positive psychosocial adjustment function is the focus of our research. Li et al. (12) reported a developmental psychopathology framework of the psychosocial need of children orphaned by HIV and demonstrated the interaction of risk and protective factors on social well-being and the dynamics of individual and environment interactions over time. Wang et al. (13) discovered that perceived social support (PSS) and trust relationships were the most proximate protective factors, and traumatic events and HIV-related stigma were a double burden for AIDS orphans. Therefore, based on a psychological adjustment development research framework and a positive psychology perspective, this study integrates the effects of risk (HIV-related stigma) and protective factors (PSS and social trust) on the positive psychosocial adjustment of youth affected by parental HIV/AIDS.

Social well-being is the self-judgment and evaluation of the relationship between oneself and others, the collective and society (14). As a “double vulnerable group,” youths affected by parental HIV/AIDS face more isolation, poverty, difficult living situations, educational disruption, HIV-related stigma, maltreatment, and hostility from families or communities than the general population (15, 16). PSS has proved to be a “buffer” against these setbacks (17). PSS refers to an individual perceptions of the general availability and quality of social support available to them, and the emotional experience generated by expectation, evaluation, and belief of social support (18, 19). The PSS is a potential protective factor for people to buffer against traumatic events and improve social well-being (10, 20). In rural China, Shan et al. (21) discovered the positive effect of PSS on psychological adjustment among children affected by parental HIV/AIDS. Hong et al. (22) found the level of PSS was significantly and positively associated with psychosocial well-being. PSS and social well-being were found to have a significantly positive relationship in adolescent studies (23, 24). Most evidence data showed that levels of PSS were the most important contributor to one’s emotional health (25). Zhao et al. (26) demonstrated a strong association between PSS and psychosocial outcomes, emphasizing the importance of adequate social support in alleviating stressful life events and improving the psychosocial well-being of children affected by HIV/AIDS in China. Based on these researches, the PSS may be an antecedent of well-being, and its availability is thought to be causal to social well-being (27). Consequently, youths affected by parental HIV/AIDS with high levels of PSS may have higher levels of social well-being.

According to the theoretical model of social well-being (14), relationships among individuals, society, and community (Social Integration) may affect social well-being by influencing whether people trust others positively (Social Acceptance). In other words, increasing PSS may increase social well-being by boosting social trust in the social environment’s security and the others’ credibility. Social trust refers to the connection network between individuals and society and the resulting norms of reciprocity and credibility (28). Wang et al. (13) found PSS impacted the trust relationships with caregivers, which in turn affected social well-being, and the trust relationships appeared to be the most proximate protective factor for social well-being among children affected by HIV/AIDS in rural China. Many studies on relevant variables provide evidence for the relationship between them. Mohanty et al. (29) found that support from the targeted community could help nurses build personal and social connections and enhance trust in the community. Strang et al. (30) found the support of families and communities was conducive to improving the trust of those becoming homeless by the conflict. Therefore, when youths affected by parental HIV/AIDS perceive more social support, they tend to have more social trust (31). The social trust may also positively predict the youths’ social well-being (32). Tokuda et al. (33), using a sample of Asian countries, found that residents of countries with a high level of social trust were happier than those with low social trust. The research in China also strongly suggested that social trust positively predicted well-being (34). In addition, “warm glow theories” pointed out that high-trust individuals were more likely to obtain additional happiness through the warm light given, indicating that higher social trust may bring better social well-being (35).

The risk factor of HIV-related stigma refers to the prejudice, discounting, and discrediting directed at people living with HIV/AIDS (PLWHA) and the groups and communities with which they are associated (36). HIV-related stigma may moderate the relationship between PSS and social trust for several reasons. The first is the appraisal and coping to the stress of HIV-related stigma, individuals who perceived or experienced high HIV-related stigma are more likely to be stressed and to have a negative appraisal and coping (37). The second reason is that HIV-related stigma makes youths affected by parental HIV/AIDS frequently feel deprived of their basic rights and social support. The unfavorable discrepancy between “value expectations (wanting)” and “value capabilities (deserving)” may decrease the effect of PSS on their social trust (38). Therefore, compared to youths affected by parental HIV/AIDS who perceived or experienced high HIV-related stigma, the effect of PSS on social trust may be stronger in those who perceived or experienced low HIV-related stigma.

At the same time, HIV-related stigma may also play a moderate role in the relationship between social trust and social well-being, but the specific moderation effect is debated. For one thing, factors in the microsystem (e.g., the stigma environment of schools and community) and macrosystem (e.g., social environment, social trust) interact to shape the developmental outcomes among youths affected by parental HIV/AIDS (39, 40). Chi et al. (8) discovered that HIV-related stigma negatively predicted healthy outcomes with considerable stability over time. Therefore, the higher HIV-related stigma to which youths affected by parental HIV/AIDS are exposed, the more likely they are to develop unhealthy outcomes, reducing the impact of social trust on social well-being. For another, different levels of social trust have a unique impact on social well-being among youths who perceived or experienced high HIV-related stigma (41). Yamamura’s et al. (42) research showed that the impact of social trust on well-being could be enhanced in natural disasters, and the enhancement effect was stronger for residents in disaster-affected areas. Studies also suggested that the uncontrollable impact in life would be alleviated with the positive impact of social trust on well-being (34). In other words, for youths who perceived or experienced high HIV-related stigma, the positive impact of social trust on social well-being may be stronger than those who perceived or experienced low HIV-related stigma. The above two inconsistent views have not been well studied in youths affected by parental HIV/AIDS yet.

**FIGURE 1:**
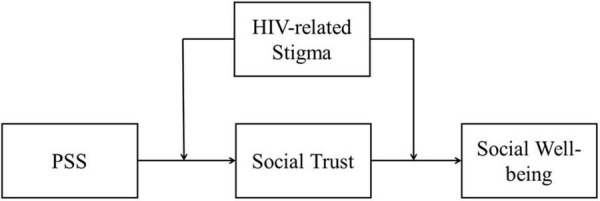
Conceptual model.

To summarize, the current study aimed to explore the relationship between PSS and social well-being of youths affected by parental HIV/AIDS, as well as the mediation effect of social trust and the moderation effect of HIV-related stigma. These aims constructed a moderated mediation model that would address how the PSS affects social well-being through social trust among youths affected by parental HIV/AIDS and when is this association most potent? (Figure 1). Based on the theoretical model of social well-being, developmental psychopathology framework of the psychosocial need, and related empirical researches, the following three hypotheses were proposed:

H1: PSS would positively predict the social well-being of youths affected by parental HIV/AIDS.
H2: Social trust would mediate the association between PSS and social well-being among youths affected by parental HIV/AIDS.
H3: HIV-related stigma would moderate the mediation model.

## Materials and Methods

### Participants

The participants were from a larger research project on the psychological adjustment of children affected by HIV, which enrolled a total of 1,625 children and adolescents aged 6–18 years in rural China, where many residents were infected with HIV in the 1990s as a result of unhygienic blood collection practices (43). We re-contacted 331 of the participants (youths affected by parental HIV/AIDS) in the prior study through local schools and the government-funded AIDS orphanages and invited them to participate in the current study. This study focuses on youths (20–30 years old) who were in the critical developmental stage of their lifetime, including those who lost one or both of their parents to HIV/AIDS and those who are living with HIV-infected parents. Youths with HIV infection were eligible to participate.

### Procedures

Data in the current study were collected in 2021 via a questionnaire survey. A mixed-method approach (online and paper-based) was employed in our survey research. We sent an informed consent and online link to the participants who worked or went to school in other places and encouraged them to participate. Because of the popularity of the internet and smartphones in China, most youths are using WeChat and Message to communicate. Therefore, an online questionnaire was distributed through WeChat and Message. After signing the consent form and reading the requirements, participants were asked to complete a comprehensive questionnaire to collect demographic information, perceived social support, perceived stigma, enacted stigma, social trust, and social well-being in order. To avoid missing data, all questions were set as required and there was no option like “not to prefer to respond” or “I do not know.” Besides, the paper-based version consistent with the online survey was mainly collected from participants who worked locally and volunteered to participate in the survey. All the participants were free to withdraw from the study at any time. There was a structured data confidentiality process in place to ensure the privacy and confidentiality of the survey. Each participant received a compensation of 50 ¥ after the survey. Among the 331 youths, 274 completed the survey online, and the response time of 12 youths was fewer than 700 s. Additional 57 participants completed a paper questionnaire, and 22 youths failed to complete the survey fully. The final sample included 297 youths (57.2% male) and the response rate was 89.7%. The research protocol, including consenting procedure, was approved by the Institutional Review Board at Henan University in China.

### Measures

#### Demographic Characteristics

Youths were asked to report on individual characteristics during the survey. These characteristics include gender, age, ethnicity, parental status (i.e., lost one or both of their parents, living with HIV-affected parents, other), work status (i.e., farming, in college, odd jobs, permanent jobs, government employees, public institution employees), current residence (i.e., countryside, town, small-medium cities, big cities), and health status (i.e., very good, good, fair, poor, very poor).

#### Perceived Social Support Scale

The perceived social support scale (PSSS) was adapted from the Multi-dimensional Scale of Perceived Social Support (MSPSS) (44). The Chinese version of the perceived social support was translated and independently back-translated from English to Chinese by Hong et al. (22). Considering the developmental stages of the participants, the parallel subscale of teacher support was removed. The modified version measures perceived social support (PSS) from three sources: family, friends, and significant others, and consists of 12 items rated on a 7-point Likert scale (from 1 = very strongly disagree to 7 = very strongly agree). The sample questions include “My family really tries to help me,” “I can count on my friends when things go wrong,” and “I have a special person who is a real source of comfort to me.” A total score was employed as a composite score for the PSSS with a higher score indicating a higher level of perceived social support. Zimet et al. (44) reported the scale has good internal and test-retest reliability as well as moderate construct validity, which has been validated in children and adolescents affected by parental HIV/AIDS (10, 22). The Cronbach’s alpha of the 12-item scale was 0.94 for this study.

#### Social Well-Being

The Social Well-being Scale (SWB) was revised by Yuanjiang and Qinghua (45) according to the social well-being scale of the Midlife Development in the United States, MIDUS (46). The modified version measures social well-being from five sources: social actualization, social coherence, social integration, social acceptance, and social contribution, and consists of 15 items rated on a 5-point Likert scale (from 1 = very inconsistent to 5 = very consistent). The sample questions include “The world is becoming a better place for everyone,” “I feel close to other people in my community,” and “My community is a source of comfort.” The total score of the scale was used with a higher score indicating a higher level of social well-being. Yuanjiang and Qinghua (45) and Keyes and Shapiro (46) reported the scale has good internal and test-retest reliability as well as moderate construct validity, which has been validated in China (45, 47). The Cronbach’s alpha of the 15-item scale was 0.93 for this study.

#### Social Trust

The Social Trust Questionnaire (STQ) was developed by Wanqing (48). The STQ measures social trust from four sources: authoritarian trust, interpersonal perception, market trust, and media identification, and consists of 17 items rated on a 5-point Likert scale (from 1 = strongly disagree to 5 = strongly agree). The sample questions include “Supermarkets (or stores) will not sell expired goods” and “More and more people keep their promises in society.” The total score of the scale was used with a higher score indicating a higher level of social trust. Wanqing (48) reported the fitting indicators were good and the internal consistency of the official scale is 0.853. All of these indicate that the self-developed social trust scale for adolescents has good reliability and validity and can be used as a tool to measure the social trust of young people. The Cronbach’s alpha of the 17-item scale was 0.92 for this study.

#### HIV-Related Stigma

##### Perceived Stigma

Perceived stigma was assessed with a 30-item scale (3, 16). This scale included three subscales. The first subscale assessed the youths’ perceptions of public stigma against HIV/AIDS patients and their living environment. Youths were asked to indicate that in their opinion how many people in the community/society would have certain stigmatizing attitudes toward HIV/AIDS patients and their families. The sample questions include “People will think someone with HIV is unclean” and “People will look down at someone who has HIV/AIDS.” The second subscale assessed the youths’ perceptions of public stigma against HIV/AIDS patients. Youths were asked to indicate in their opinion whether they agreed with some attitudes toward HIV/AIDS patients in society. The sample questions include “AIDS patients should be ashamed of themselves” and “AIDS patients should be isolated.” The third subscale assessed the youths’ perceptions of public stigma against youths affected by parental HIV/AIDS. Youths were asked to indicate that in their opinion how many people in the community/society would have certain stigmatizing attitudes toward them. The sample questions include “People think youths affected by parental HIV/AIDS should leave their villages” and “People think youths affected by parental HIV/AIDS are unclean.” All items were reverse-scored when appropriate to have higher total scores suggesting higher levels of perceived stigma. Zhao et al. (16) reported the scale demonstrated good content validity, which has been validated in children and adolescents affected by parental HIV/AIDS (3, 9, 16). The Cronbach’s alpha of the 30-item scale was 0.94 for this study.

##### Enacted Stigma

Enacted stigma consists of 14 items rated on a 5-point Likert scale (from 1 = never happened to 5 = always happened) (16), youths affected by parental HIV/AIDS were asked to indicate whether they had experienced any stigmatized actions after a parental HIV infection. Sample items included “Being beaten by others” and “Being called bad names.” The total score of the scales with a higher score indicates a higher level of enacted stigma. Previous studies reported the scale demonstrated good content validity, which has been validated in children and adolescents affected by parental HIV/AIDS (3, 9, 10, 16). The Cronbach’s alpha of the 14-item scale was 0.95 for this study.

### Statistical Analysis

Data were analyzed with IBM SPSS Statistics (Version 25) in four steps. First, descriptive statistics were employed to display demographic characteristics of the sample. Measurement data were presented as (mean ± standard deviation), and the results showed that the data were normally distributed. Second, the degree of common method deviations in the data was tested using the Harman one-factor model method. Third, the bivariate correlations among key study variables were measured with Pearson’s correlation coefficient. Fourth, the present study conducted multiple regressions using PROCESS Marco (Model 59) to analyze the moderated mediation effect. Before regression analyses, all variables except gender were standardized. In regression Model 1, PSS, enacted stigma, and the interaction of PSS and enacted stigma were predictor variables, social trust was outcome variable; In regression Model 2, PSS, perceived stigma, and the interaction of PSS and perceived stigma were predictor variables, social trust was outcome variable; In regression Model 3, PSS, social trust, enacted stigma, and the interaction of social trust and enacted stigma were predictor variables, social well-being was outcome variable; In regression Model 4, PSS, social trust, perceived stigma, and the interaction of social trust and perceived stigma were predictor variables, social well-being was outcome variable. Gender and age were the control variable in all regression models. A simple slopes analysis was also carried out in the PROCESS Macro to examine the nature of these moderating effects.

## Results

### Sample Characteristics

Table 1 outlines demographic characteristics of the 297 participants (57.2% male) including group, gender, age, work status, current residence, and health status. The participants included 43.40% (*n* = 129) orphans and 56.60% (*n* = 168) vulnerable youths. The mean age of the sample was 25.80 years (SD = 3.14), with a range from 22 to 29 years. About 45.8% of youths lived in the countryside, 46.4% have relatively stable jobs (37.4% permanent jobs, 1.3% government employees, and 7.7% public institution employees) and 84.20% have very good (61.30%) or good (22.9%) good health status.

**TABLE 1:** Background characteristics of the HIV-affected youths (*N* = 297).

Characteristics	*n*	%
**Group**		
AIDS orphans	129	43.40
Vulnerable youths	168	56.60
**Gender**		
Male	170	57.20
Female	127	42.80
Age (*M* ± *SD*)	25.80 ± 3.14	
**Work status**		
Farming	48	16.20
In College	43	14.50
Odd jobs	68	22.90
Permanent jobs	111	37.40
Government employees	4	1.30
Public institution employees	23	7.70
**Current residence**		
Countryside	136	45.80
Town (county seat and below)	50	16.80
Small-medium cities	57	19.20
Big cities	54	18.20
**Health status**		
Very good	182	61.30
Good	68	22.90
Fair	37	12.50
Poor	9	3.00
Very poor	1	0.30

*AIDS orphans, orphans who lost one or both of their parents to AIDS; Vulnerable youths, youths who were living with HIV-infected parents.*

### Testing of Common Method Deviations

The data collected in this study were collected from the self-reports of the respondents, and thus common method deviations may exist. Statistically, the results showed that there were 15 factors with eigenvalues greater than one, and the first factor explained only 23.85% of the variance, which was much less than the critical value of 40%, indicating that there were no serious homoscedasticity deviations for each variable in this study.

### Preliminary Analyses: Correlation

The mean, standard deviation, and Pearson correlation coefficient for each variable were presented in Table 2. The results showed that the perceived social support (PSS) was significantly positively correlated with social well-being (*r* = 0.43, *p* < 0.001) and social trust (*r* = 0.46, *p* < 0.001), and significantly negatively correlated with perceived stigma (*r* = −0.37, *p* < 0.001) and enacted stigma (*r* = −0.29, *p* < 0.001). Social well-being was significantly positively correlated with social trust (*r* = 0.67, *p* < 0.001), and significantly negatively correlated with perceived stigma (*r* = −0.28, *p* < 0.001). Social trust was significantly negatively correlated with perceived stigma (*r* = −0.34, *p* < 0.001) and enacted stigma (*r* = −0.24, *p* < 0.001). The perceived stigma was significantly positively correlated with enacted stigma (*r* = 0.30, *p* < 0.001).

**TABLE 2:** Means, standard deviations, and correlations of variables (*N* = 297).

	*M*	*SD*	1	2	3	4	5	6
1. .Gender^a^	−	−	1					
2. .Age	25.80	3.14	−0.04	1				
3. .PSS^b^	61.34	13.99	0.00	0.11	1			
4. Social well-being	57.33	10.15	−0.01	0.11	0.43***	1		
5. .Social trust	56.21	11.55	−0.06	0.02	0.46***	0.67***	1	
6. .Perceived stigma	64.44	16.72	−0.08	0.04	−0.37***	−0.28***	−0.34***	1
7. .Enacted stigma	21.91	9.73	−0.08	−0.06	−0.29***	−0.08	−0.24***	0.3***

*^a^0, boys; 1, girls. ^b^PSS, perceived social support.*

***p < 0.001.

### Testing for Moderated Mediation Effect

**FIGURE 2:**
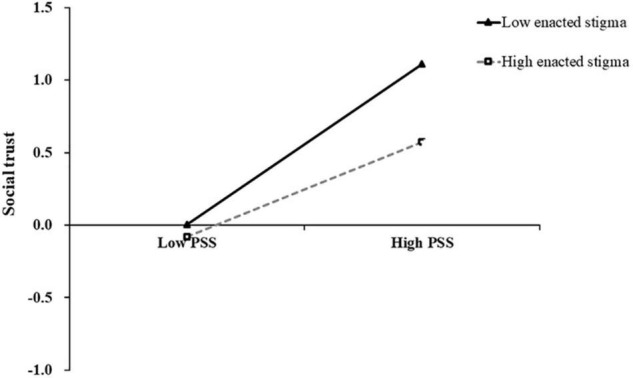
The moderating effect of enacted stigma on the relationship between PSS and social trust.

All predictor variables were standardized in each equation, controlling for key demographic variables such as age and gender. As shown in Table 3, PSS significantly positively predicted social well-being (β = 0.19, *p* < 0.001) and social trust (β = 0.43, *p* < 0.001). Social trust positively predicted social well-being (β = 0.60, *p* < 0.001). Besides, the interaction terms of PSS and enacted stigma were also significant in predicting social trust (β = −0.11, *p* = 0.03), but the interaction terms of social trust and enacted stigma were not significant in predicting social well-being (β = 0.03, *p* = 0.50). To reveal how the interaction terms of PSS and enacted stigma predicted the social trust, we grouped high and low according to the score of enacted stigma and plotted the interaction as shown in Figure 2. The simple slope test (simple slope test) showed that when the level of enacted stigma was low (−1 SD), the degree of PSS on social trust showed a more obvious upward trend (*bsimple* = 0.54, *t* = 7.12, *p* < 0.001). When the level of enacted stigma was high (+1 SD), the degree of PSS on social trust showed an upward trend with signs of leveling off (*bsimple* = 0.33, *t* = 4.66, *p* < 0.001). Thus, the positive influence of PSS on social trust was moderated by the enacted stigma.

**TABLE 3:** Moderated mediation effects test for HIV-related stigma.

Regression equation (*n* = 297)			Overall fitting index		Significance of regression coefficient		
		
Outcome variable		Predictor variable	*R* ^2^	*F*	β	*t*	95% CI
Model 1	Social trust	PSS	0.24	18.49***	0.43	8.03***	[0.33,0.54]
		Enacted stigma			−0.16	−2.93**	[−0.27, −0.05]
		PSS × Enacted stigma			−0.11	−2.17*	[−0.20, −0.01]
		Gender			−0.14	−1.33	[−0.34, 0.07]
		Age			−0.01	−0.58	[−0.04, 0.02]
Model 2	Social trust	PSS	0.25	19.84***	0.38	7.00***	[0.28, 0.49]
		Perceived stigma			−0.21	−3.75***	[−0.31, −0.10]
		PSS × Perceived stigma			−0.08	−1.54	[−0.17, 0.02]
		Gender			−0.16	−1.60	[−0.37, 0.04]
		Age			−0.01	−0.37	[−0.04, 0.03]
Model 3	Social well-being	PSS	0.49	40.06***	0.19	3.83***	[0.09, 0.29]
		Social trust			0.60	12.15***	[0.50, 0.70]
		Enacted stigma			0.10	2.07*	[0.00, 0.19]
		Social trust × Enacted stigma			0.03	0.67	[−0.05, 0.11]
		Gender			0.10	1.22	[−0.06, 0.27]
		Age			0.03	1.98*	[0.00, 0.05]
Model 4	Social well-being	PSS	0.48	38.07***	0.14	2.76**	[0.04, 0.24]
		Social trust			0.58	11.83***	[0.49, 0.68]
		Perceived stigma			−0.06	−1.22	[−0.15, 0.04]
		Social trust × Perceived stigma			0.10	2.04*	[0.00, 0.19]
		Gender			0.06	0.74	[−0.11, 0.23]
		Age			0.03	1.93	[−0.00, 0.05]

*p < 0.05, **p < 0.01, ***p < 0.001. PSS, perceived social support.

**FIGURE 3:**
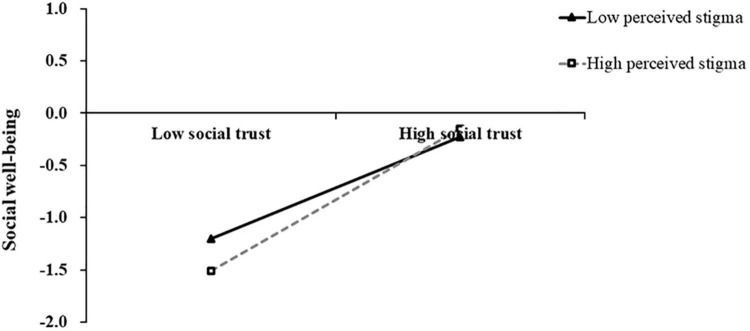
The moderating effect of perceived stigma on the relationship between social trust and social well-being.

The same procedure was used to test the effect of perceived stigma. As shown in Table 3, PSS significantly positively predicted social well-being (β = 0.14, *p* = 0.01) and social trust (β = 0.38, *p* < 0.001). Social trust positively predicted social well-being (β = 0.58, *p* < 0.001). The interaction terms of social trust and perceived stigma were also significant in predicting social well-being (β = 0.10, *p* = 0.04), while the interaction terms of PSS and perceived stigma were not significant in predicting social trust (β = −0.08, *p* = 0.12). To reveal how the interaction terms of social trust and perceived stigma predicted social well-being, we grouped high and low according to the score of perceived stigma and plotted the interaction as shown in Figure 3. The simple slope test (simple slope test) showed that when the level of perceived stigma was high (+1 SD), the degree of social trust in social well-being showed a more obvious upward trend (*bsimple* = 0.68, *t* = 10.12, *p* < 0.001). When the level of perceived stigma was low (–1 SD), the degree of social trust on social well-being showed an upward trend with signs of leveling off (*bsimple* = 0.49, *t* = 6.97, *p* < 0.001). Thus, perceived stigma could play a moderated role in the positive effect of social trust on social well-being.

## Discussion

This study found that perceived social support (PSS) could significantly predict social well-being among youths affected by parental HIV/AIDS, the result was consistent with previous research findings (26, 49) and confirmed the hypothesis of H1. Researchers believed the social support “buffered” (protected) youths affected by parental HIV/AIDS from the potentially pathogenic influence of stressful events and could provide a positive effect, recognition of self-worth, and the ability to integrate social networks (50). Youths who have high levels of PSS are more likely to integrate social networks (51). They may maintain external social support and strengthen protective factors against negative social challenges, implying they may integrate more support from society (51, 52). Nyoni et al. (49) found that PSS and family cohesion protected the psychological well-being of youths affected by parental HIV/AIDS, implying youths who have high levels of PSS may maintain more support from family and relatives. Studies also showed that youths who have more friends’ or colleagues’ support may develop physical function, social function, and cognitive function well, and improve their social well-being, implying people who have high levels of PSS may maintain more support from friends or colleagues (53, 54). Therefore, the youths affected by parental HIV/AIDS who have high levels of PSS may transform the external social support into the internal resources to deal with life events and have a high level of social well-being.

Consistent with hypothesis H2, the results showed that social trust played a mediating role between PSS and social well-being, which was consistent with the previous research results (35) and verified again that trusting relationships were the most proximate protective factor for social well-being (13). For the first stage, PSS may affect the cognitive evaluation of environmental adaptability of youths affected by parental HIV/AIDS. Individuals with high levels of PSS have a strong sense of belonging, obligation, care, respect, and intimacy (55, 56). The sense of social belonging means being accepted and tolerated by other group members (57) and means having a better relationship with group members. The better the relationship between individuals and society, the higher the level of social trust (46). In the second stage, social trust is related to beneficial interpersonal relationships and social care, which plays a positive role in promoting social well-being (58). Research has found that individuals with high levels of trust will have higher social cohesion and well-being in society (59). Carattini and Roesti (35) also pointed out that people who have high levels of social trust were more likely to feel the warm light given, realize their value, and feel more well-being. As far as this study is concerned, the PSS significantly increases social well-being via increasing social trust among youths affected by parental HIV/AIDS.

In line with our hypothesis H3, the results showed that different types of HIV-related stigma (perceived stigma and enacted stigma) had different moderated effects on the mediatory model, it found new different pathways of perceived stigma and enacted stigma influenced social well-being of youths. Specifically, enacted stigma moderated the relationship between PSS and social trust. This study found that the effects of PSS on social trust were stronger among youths who perceived or experienced low enacted stigma than those who perceived or experienced high enacted stigma. The result was consistent with the developmental psychopathology framework of psychosocial need (12). Individual risk factors (enacted stigma) will weaken the beneficial impact of PSS on social trust (60, 61). The more personal risk factors (enacted stigma) happened to youths affected by parental HIV/AIDS, the more they feel that they aren’t protected or accepted in interaction with the social environment, and the more difficult for them to establish trusting interpersonal relationships with others. Researchers suggest that the brain expects to establish harmonious social relations including mutual trust and interdependence. If these expectations are not met, the brain will perceive less positive resources and more stress, and increase more negative attitudes toward the external environment (62, 63). Youths who perceived or experienced more enacted stigma are more likely to have a negative attitude toward the external environment. Therefore, when the level of enacted stigma is high, the beneficial impact of PSS on social trust will be significantly reduced.

In addition, perceived stigma moderated the relationship between social trust and social well-being. Our data showed that the positive predictive effect of social trust on social well-being was stronger in youths who perceived more stigma. In specific, the development disadvantages of those who perceived high stigma are more reflected in the situation of low social trust. Increasing social trust may benefit those who perceived high stigma (60), which was inconsistent with the negative impact of perceived stigma among children affected by parental HIV/AIDS (9). The result supported that the children’s perceived stigma was linearly decreased with age, the linear age trend of perceived stigma may reflect the relative maturity in cognition and emotion among children as they mature (36). Internal resources (mature cognitive capacity and coping skills) together with relational (trusting relationships) and community resources (supportive environment) were suggested to be associated with better neurobiological and psychosocial outcomes (64). Other studies had shown that in a chaotic environment, social trust would play a greater role, and the relationship between social trust and well-being would become stronger after a disaster (42). Youths who perceived high stigma may suffer more psychological disasters than those who perceived low stigma. They are more likely to be influenced by social trust-related interventions. The moderation effect also shows that youths affected by parental HIV/AIDS need more social warmth and concern to improve their social well-being.

## Limitations

Several study limitations should be noted. First, because the HIV epidemic in the research site included was primarily due to unhygienic blood collection, the sample size of this study is relatively small and may not be representative of all youths affected by parental HIV/AIDS in other settings. Second, an online survey may exist the possibility of a selection bias and cannot control for the effects of some additional variables, which was affected by the participant’s cooperation attitude. Furthermore, the data was gathered through self-report, which may be subject to social desirability and self-reporting bias. Third, confounding variables caused by inconsistent environmental variables may affect findings among youth affected by AIDS, and the cross-sectional data in this study can only reveal the correlation among all variables. Thus, future research can include more types of youths affected by parental HIV/AIDS, and combine the evidence of multiple disciplines (psychophysiology, brain science, etc.) and multiple indicators (behavioral experiments, brain imaging, etc.) to reveal the role of perceived social support (PSS) and the occurrence mechanism of HIV-related stigma among vulnerable groups, as well as the causal relationship among variables.

## Conclusion

This study constructed a moderated mediation model to explore whether HIV-related stigma depresses their social well-being, discovering the risk and protective factors for social well-being, as well as the unique contribution of different types of HIV-related stigma. The findings are crucial for intervention and social well-being enhancement among youths affected by parental HIV/AIDS. First, future health promotion and psychological care efforts for youths affected by parental HIV/AIDS must consider the effect of various forms of HIV-related stigma on these youths’ social well-being. Second, as social trust is the most proximate protective factor for social well-being, it should be a focal point and an important goal for future prevention interventions aimed at improving social well-being among youths affected by parental HIV/AIDS. Finally, intervention programs should be developed by the government, society, community, health care settings, and practitioners to mitigate the negative effect of risk factors (HIV-related stigma) and promote protective factors (PSS and social trust).

## Data Availability Statement

The datasets presented in this article are not readily available because the data involving special minority participants must be kept confidential. Requests to access the datasets should be directed to JZ, jfzhao63@hotmail.com.

## Ethics Statement

The studies involving human participants were reviewed and approved by the Institutional Review Board at the Henan University in China (IRB 00007212). The patients/participants provided their written informed consent to participate in this study.

## Author Contributions

YZ conceptualized the manuscript, performed statistical analyses, and wrote the first draft of the manuscript. LJ, GL, YS, and XL revised the manuscript for important intellectual content. JZ guided and supervised the whole study. All authors reviewed and approved the final version of the manuscript.

## Conflict of Interest

The authors declare that the research was conducted in the absence of any commercial or financial relationships that could be construed as a potential conflict of interest.

## Publisher’s Note

All claims expressed in this article are solely those of the authors and do not necessarily represent those of their affiliated organizations, or those of the publisher, the editors and the reviewers. Any product that may be evaluated in this article, or claim that may be made by its manufacturer, is not guaranteed or endorsed by the publisher.

## Abbreviations

PSS: perceived social support
PSSS: perceived social support scale
SWB: social well-being
PLWHA: people living with HIV/AIDS.
